# Transitioning to life after sport: empowering former college varsity athletes to live more healthfully

**DOI:** 10.3389/fspor.2025.1713432

**Published:** 2026-01-12

**Authors:** Linda B. Piacentine, Taylor L. Wolf, Jacob J. Capin

**Affiliations:** 1 Athletic and Human Performance Research Center and College of Nursing, Marquette University, Milwaukee, WI, United States; 2 Life After Sport Trajectories (LAST) Lab, Department of Physical Therapy, Marquette University, Milwaukee, WI, United States; 3 Medical College of Wisconsin, Clinical and Translational Science Institute of Southeast Wisconsin, Milwaukee, WI, United States

**Keywords:** college athlete, lived experience, long-term health, qualitative study, sport transition

## Abstract

**Introduction:**

The transition out of competitive sport comes with a range of psychosocial challenges for athletes, particularly when retirement is involuntary (e.g., due to injury). Little is known about the health of former athletes who often manage prior injuries and changing biopsychosocial factors.

**Purpose:**

Identify the unique experiences of varsity athletes during college and as they transition to life after college sports and determine the facilitators, barriers, and needs of college varsity athletes to engage in a physically active and healthy lifestyle after competitive sport retirement.

**Methods:**

Thirty former college varsity athletes (15F, 15M; mean age 23 ± 1 yrs; BMI: 26 ± 4 kg/m^2^) who finished competing approximately 2 weeks to 2 years prior participated in semi-structured, qualitative interviews asking open-ended questions describing their experiences as college athletes and their transition to life after college sports. Interview topics centered on physical and psychological health, physical activity and exercise, diet, the overall transition experience including ways in which athletes felt well equipped or poorly equipped for life after sport, and what could have facilitated their transition to life after collegiate athletics. Researchers conducted coding and thematic analysis using an iterative and collaborative approach until no new themes were constructed.

**Findings:**

Most athletes participated in National Collegiate Athletic Association (NCAA) Division I (87%) or III (10%) sports. Athletes represented a variety of sports with Volleyball (*N* = 7), Soccer (*N* = 6), and Track & Field (*N* = 6) more common than others. Three main themes were constructed: (1) College athlete uniqueness; (2) Transitioning to life after college sport; and 3) Empowering former athletes to live more healthfully. Despite many unique and beneficial experiences of college athletes, the highly scheduled lives of college athletes led to a post-retirement loss of structure and support, negatively impacting those without a clear post-college sports plan and social network. Former athletes described pain and soreness that often lessened or resolved after a few months of no longer competing in sports. Athletes expressed how they could have been empowered to live more healthfully in the transition away from college athletics including guidance on nutrition, managing prior injuries, and exercising for general health rather than sports performance.

**Discussion:**

Competitive athletes have unique experiences that both equip and challenge them as they transition away from structured sport environments and the associated support systems. Former athletes identified several key factors that may facilitate a healthier transition, including guidance on exercising for general health, managing pain and prior injuries, nutrition, and social support. Understanding the needs of athletes transitioning out of competitive sport will better equip healthcare providers to counsel, educate, and treat athletes for optimal long-term health.

## Introduction

While historical data suggest former elite athletes may be fitter and healthier than their nonathlete counterparts (1), contemporary evidence challenges this notion (2, 3). Prior participation in high-level, competitive sport alone does not guarantee long-term health, and former athletes must continue to be physically active to preserve body composition, function, and cardiovascular health (2). Some former athletes continue to be active while others do not (2, 4, 5). Findings from Simon and Docherty indicate that midlife former college athletes have poorer functional performance and fitness (3), higher body fat (3), and steeper declines in self-reported physical function and pain 5 years later compared with nonathletes (6) but these studies did not account for prior injuries which are high among former athletes. A significant need exists to investigate the transition out of competitive sport and develop strategies to support contemporary competitive athletes during this critical period.

The transition out of competitive sport comes with a range of psychosocial experiences for athletes and is particularly challenging when retirement is involuntary (e.g., due to injury) (7–9) and/or a surprise (10, 11). A topical review by Moore et al. (8) emphasized the importance for athletes to be adequately prepared for sport retirement, although this review of qualitative studies investigating the biopsychosocial experiences of elite athletes retiring from sport after career-ending injuries found only 3 relevant studies consisting of 7–10 participants each. Their key findings were that elite athletes retiring from sport after career-ending injuries often are challenged by a loss of athletic identity, a lack of external support, and a decline in mental health; and that social support may mitigate these challenges (8). Qualitative and mixed-methods studies on the transitional experiences of retiring athletes often focus on psychosocial and/or mental health barriers and challenges (11–14). Less attention has been paid to the intersection of mental health with physical health of athletes and how the two are intertwined in the college varsity athlete during and after retirement from sport.

Considerably less is known about the physical fitness and overall health experiences of former athletes who often manage prior injuries and changing biopsychosocial factors. Some scholars and athletes even question whether the focus of sport is ever truly on overall health (15). A recent study by Papathomas et al. in 31 retired elite athletes, many of whom were Olympians, highlighted the significant challenges many athletes face accepting inevitable post-retirement physical health and body image changes and their difficulties adjusting to regular eating and exercise routines with inadequate support (16). Thibodeau and colleagues identified sociocultural factors related to body dissatisfaction and disordered eating in retired elite women athletes (17). A case series of two former college football players, one of whom trained for the National Football League (NFL) draft and the other who stopped training for football, noted more pronounced negative health changes in the former college football player who stopped training (18). Our recently published qualitative study in midlife former college athletes indicates that athletes describe many benefits from their participation in competitive sports yet note challenges transitioning out of competitive sport including managing pain, prior injury, and changing energy intake demands (4).

Our ongoing, mixed methods clinical study is investigating the impact of college sport participation and musculoskeletal injury on long-term health outcomes to help address this void. Our work addresses several of the recommendations proposed by Reifsteck and colleagues (19) for sports medicine professionals to promote student-athlete well-being during the transition from collegiate sport. Determining the barriers and needs of contemporary college athletes who are navigating the transition out of competitive sports will better equip healthcare providers to counsel, educate, and treat athletes for optimal long-term health.

### Research questions

The overarching research questions were: (1) What are the experiences of college varsity athletes as they transition to life after sport? and (2) What could facilitate the transition to life after college athletics to improve short- and long-term health?

### Objectives of the study

The purpose of this study was to (1) identify the unique experiences of varsity athletes during college and as they transition to life after college sports; and (2) determine the facilitators, barriers, and needs of college varsity athletes to engage in a physically active and healthy lifestyle after competitive sport retirement.

## Methods

### Study design

This qualitative study is from an ongoing parent clinical mixed-methods study investigating the impact of college sport participation and musculoskeletal injury on long-term health outcomes (NCT05344001). A qualitative description methodology keeps the researchers close to the data with lower amounts of interpretation than other qualitative methods (20). In this methodology, the researchers return to the data repeatedly while coding to keep the entire text in mind while identifying the themes. This methodology does not start with a theory but rather uses an inductive approach which acknowledges the expertise of researchers in constructing new knowledge. The methodology draws on the strengths of researchers' experiences when interpreting and summarizing the data (20). In this case, our interdisciplinary team included two former collegiate athletes and physical therapists (JJC and TLW) and a qualitative nurse researcher (LBP). The study was approved by the Marquette University IRB (IRB# 3967).

### Participants

Participants were 30 former college varsity athletes who were interviewed between April 2022 and December 2024 (Table 1). All participants spoke English. Enrollment eligibility included having previously participated in a collision, contact, and/or jumping, cutting, or pivoting sport (e.g., basketball, football, soccer, softball, volleyball) at the collegiate varsity level. Study exclusion criteria were: neurologic (e.g., stroke, Parkinson's) and/or degenerative disease that impairs function; pregnancy; and lower extremity joint replacement. Young adults who enrolled in our ongoing mixed-methods clinical study were invited to participate in interviews (described below) if they were no longer participating in college varsity sports, having completed their college varsity athletics eligibility or having no intention to participate in college sports in the future. Initially, all eligible individuals who enrolled in the parent study were invited to participate in this qualitative study during their baseline or annual follow-up visit after they had retired from sport. As individuals enrolled in this qualitative study, the research team used purposive sampling to ensure participants represented a variety of sports and to balance enrollment by sex with a target recruitment goal of 15 males and 15 females. To protect confidentiality, the school(s) where each athlete completed their college varsity sports were not recorded. All participants provided written informed consent prior to participating in this study.

**Table 1:** Descriptive characteristics of the research participants.

Outcome variable	Descriptive statistics: mean ± SD or *N* (%)
Age (years)	23 ± 1 (range: 22–25)
Sex	Male: 15 (50%); Female: 15 (50%)
Body Mass Index (Kg/m^2^)	25.8 ± 4.2 (range: 19.9–38.1)
College Sport Division (Highest Level)	NCAA D1: 26 (86.7%), NCAA D2: 0 (0%), NCAA D3: 3 (10%), NAIA: 1 (3.3%)
Primary College Varsity Sport^a^	Baseball: 1 (3.3%), Basketball 1 (3.3%), Track & Field: 6 (20%), Football: 2 (6.7%), Lacrosse: 3 (10%), Rugby: 1 (3.3%), Soccer: 6 (20%), Softball: 1 (3.3%), Tennis: 2 (6.7%), Volleyball: 7 (23.3%)

SD, standard deviation; N, number; NCAA, national collegiate athletic association; D, division (e.g., D1 is division 1); NAIA, national association of intercollegiate athletics.

a One athlete participated in two college sports: lacrosse and hockey.

### Qualitative data collection

Participants completed in-person, semi-structured interviews that were previously described in detail by Capin et al. (4). Participants were asked to describe their experiences as college athletes and their transition to life after college sports. Interview topics centered on physical and psychological health, physical activity and exercise, diet, the overall transition experience including ways in which athletes felt well equipped or poorly equipped for life after sport, and what could have facilitated their transition to life after collegiate athletics. The interview guide was published previously [see (4, Supplementary Table S1)]. Briefly, interviews were conducted in a private room, led by the first and/or last author, and averaged 30 min in length (standard deviation: 8 min; range: 16–54 min). One or two additional research team members were typically present. Interviews were audio-recorded and transcribed verbatim using transcription software (otter.ai) and reviewed for accuracy. Participants were instructed to not provide identifying information; however, in rare instances where participants said identifying information for self or others or their college attended, the identifying information was redacted.

### Reflexive thematic analysis

Following data collection, research team members reviewed and coded transcripts using an iterative process aligning with reflexive thematic analysis (21–24) which was completed using Dedoose (25). An initial code list reflective of the interview questions was developed. Research team members met frequently to discuss the code list, add new codes, and combine previous codes into themes and subthemes. Once initial themes were generated, the research team continued to review and reflect on the coding and themes until the team reached full consensus on the constructed themes and subthemes. During the process, any conflicts on the nuanced meanings of the themes were resolved via discussion of all team members. Once the codes were agreed upon, the list was applied to all the previously coded transcripts. All transcripts were reviewed by at least two team members to interpret the data from different perspectives. Data were reviewed in each of the supporting codes and summarized, and themes were generated and supported with the analyzed data.

### Quantitative data collection and analysis

Several other metrics were captured to describe the study participants. Participants self-reported their sex, race, and ethnicity. Height was assessed in centimeters using a stadiometer and weight was measured in kilograms using a scale. Body mass index (BMI) was calculated using the measured height and weight values. Participants completed a detailed sports history survey with a trained study team member that included their college varsity sport(s) and highest level of collegiate competition (e.g., Division).

## Findings

**Figure 1:**
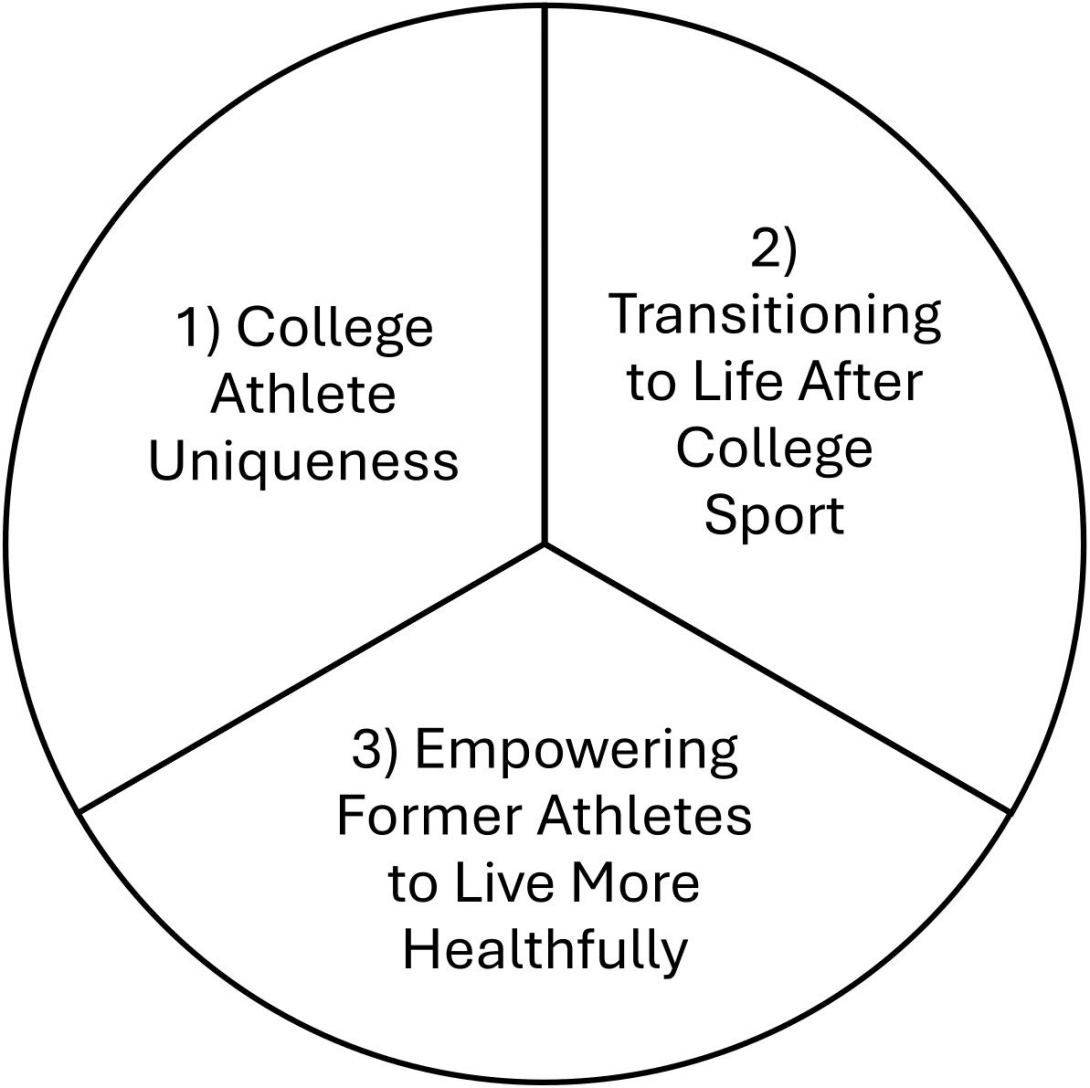
Three main themes.

Three major themes were constructed (Figure 1): (1) College athlete uniqueness; (2) Transitioning to life after college sport; and (3) Empowering former athletes to live more healthfully. Several subthemes were also developed within each of the themes (Table 2).

**Table 2:** Primary themes with associated subthemes.

Primary themes	Subthemes
Theme 1: College athlete uniqueness	• Physical health as a student-athlete ◦ Physical activity and exercise ◦ Nutrition ◦ Pain ◦ Sports injury • Identity • Intersection of mental health and sports ◦ Psychological support ◦ Emotions • Burnout • Connections and impact of coaches • Skills and characteristics of student-athletes • Changed routine: COVID-19 effects
Theme 2: Transitioning to life after college sport	• Changing identity • Loss of structure • Physical health during the transition ◦ Feeling better ◦ Managing prior injuries during the transition • Exercise during the transition • Nutrition during the transition • Intersection of mental health and life post-college sport ◦ Psychological support ◦ Feelings after sport
Theme 3: Empowering former athletes to live more healthfully	• Resources to facilitate transition • Career planning • Advice

### Theme 1: College athlete uniqueness

Understanding the unique experiences of varsity athletes during college is essential to create a foundation for understanding the transition to life after college sports including the benefits, challenges, and opportunities that former college varsity athletes face as they transition away from collegiate athletics. Several subthemes were constructed from the qualitative data that illustrate how college varsity student-athletes have very different experiences during college than their college student counterparts who do not participate in varsity athletes. College athletes live more scheduled and structured lives that can be demanding and rewarding, humbling and glorifying, socially rich and supportive yet uniquely challenging, and filled with perks and pressures that few others face. These unique experiences shape a framework that has long-lasting implications (both positive and negative).

#### Physical health as a student-athlete

Participants discussed a mixture of experiences and expressions of physical health as student-athletes. Physical activity and exercise along with nutrition, pain, and sports injuries were key components to physical health (Table 2). While athletes discussed exceptional fitness and performance, many noted significant pain and soreness even in the absence of an injury limiting their ability to participate in sports. Some noted needing to gain weight (e.g., beefing up for football) whereas others cut weight to the point where female athletes routinely lost their period during the season. Resources including access to a dietician or team chef varied widely among athletes on different teams and from different institutions and divisions.

Former athletes noted they often pushed through significant fatigue, pain and soreness throughout their sport careers. Experiences of fatigue included daily wear and tear that accumulated as the season progressed.

I never had any, any real injuries, but it was just, you could just kind of feel it every day … that end of season piece was tough. You know, you could feel just heavy. Legs were heavy … My body kind of needs the break. (ID# 1153)

Many, though not all, felt much better after several weeks to months of no longer training and competing at a high level. One noted, “*When I'm in season, I was just always fatigued and tired*” (ID# 1002). These feelings often resolved or lessened during the off-season.

Former athletes mentioned ignoring minor pain as they wanted to continue to compete despite pain. Others dismissed the daily pain as they had a prior injury and “*after just going through that injury, I really know what true pain is*” (ID# 1088). One athlete described the “athlete pain mentality” with the difference between “good” vs. “unhealthy pain” with the former due to hard sports training and the latter due to injury:

Putting that athlete mentality first. The pain is obviously part of it. It's hard to think as an athlete [you] know what is unhealthy pain and good pain, because you're in the weight room, or you're running, you know, 800 [meter] repeats with I don't know, 30 seconds rest in between you run another one, another one, another one. And so that is really, really, really painful. And so where do you draw the line between that to hit painful when you love it, and you're seeing results on track and you're getting faster? And then okay, I'm in pain when I'm walking around, but I'm still getting faster on the track so is this pain that I'm having in my day-to-day life okay because I'm still getting to where I want to be. So yeah, it's really complicated, I think. You're definitely an athlete first in college. (ID# 1076)

Sports injury could lead to separation from the team which was closely intertwined with pain and mental health concerns, including concerns about the future and the ability to become a professional player.

It was hard [to be permanently benched due to repeated concussions]. It was hard to see my friends, my two roommates still play. And when they leave to go to practice, I should be getting up too and going with them. But I don't have to. But I think it's nice for me, because I'm still around enough that I can still get the benefits of the friendship of the team and all that stuff without necessarily having to show up every day and you know, be in pain and be sore and tired and sweaty in class. (ID# 1058)

Sports injury also led to concerns about lifestyle limitations: “*She [a friend and fellow athlete] told me, “I'm super worried, like, am I gonna be able to hold my kid later in life and not have pain?”*” (ID# 1076).

#### Identity

Many athletes talked about how being in sport takes over their life, often becoming their entire identity in childhood. Being an athlete led to many commitments including team meetings, practices, and events such as travel and competition. Several athletes mentioned finding ways to prolong their time in sports through changing colleges or choosing an athletic oriented career to keep them close to sports.

I think I was realizing that my identity as an athlete was going to kind of go away, but I wasn't ready to leave the sports world yet. And so, I kind of chose to go into a profession to stay in that world. And in some capacity, even if it wasn't being an athlete, specifically. (ID# 1096)

#### Intersection of mental health and sports

Athletes expressed the need for social and/or psychological support. Some athletes talked about coming to college with recognition as great high school athletes, and the difficult change when they entered a team with others who excelled. They appreciated talking to other athletes with the same experience of joining a more skilled group. Peer athlete support groups which included mindfulness training during sport also were beneficial. One athlete commented “*those [peer support groups] were super helpful just to talk to other student athletes going through the same grind that I was going through*” (ID# 1003).

Mental health was impacted by positive and negative stress felt as a student-athlete. For some the sport participation helped in coping with stress: “*sometimes the academics put me under stress. So, I had this release of stress when I was going to practice every day*” (ID# 1008). For others, the sport participation was a time to not think about the upcoming test or difficult life events. After family tragedies, one athlete found sport was a distraction and when he finished playing, he noticed “*these things [tragedies] are all like coming to the surface a lot more*” (ID# 1157).

On the other hand, some found sport participation created substantial stress related to performance and could impact health. One athlete described losing 20 pounds due to “*worrying about getting in trouble or not performing the best or being responsible for letting the team down was always the mentality whether it was at practice or missing practice or getting sick or?*” (ID# 1079).

#### Burnout

Some athletes encountered stress to the point of feeling exhausted mentally, emotionally, and physically leading to feelings of burnout. One described how seniors and fifth year students struggled to balance internships with sport and started to “*burnout and check out*” (ID# 1078) contributing to her own feelings of being glad that sports was ending. Another athlete noted the extreme pressure of NCAA Division I sport: “*they work [you] beyond your limits. And going beyond your limits—it's always good for growth, but it comes to a point where it pushes you too much and you start to feel a little burnout*” (ID# 1016). Another was more emphatic:

I lost a lot of friends. They didn't die; they just quit. They burnt out, they washed out of the program because they couldn't take the intensity that we were training at. And they just stopped caring. They were on a scholarship and my coach kind of made it clear that they could just be managers. (ID# 1035)

Athletes have unique needs for mental health support that addresses their joining a skilled group of athletes and dealing with the stressors of being in a team sport. Burnout in a sport can lead to the desire for separation from sport but is complicated by the presence of athletic scholarships which may be lost.

#### Connections and impact of coaches

Many athletes talked about the value of their teammates, coaches, and support staff, emphasizing the connections they made both on the team and meeting people beyond the team through sports.

My teammates really did turn into my family. And they were the ones that helped me get up every day and show up at 6 AM lift for myself. And for them, you know, because again, collegiate athletics is really difficult, regardless of what sport you do at what time in your life … to be able to kind of lean on your teammates in times of need was really important to me. And that's one thing I am going to miss so much is having those people by my side that I could call at three in the morning. (ID# 1043)

Coaches could be tremendous connections and role models as supportive coaches “*cared about all of us*” (ID# 1035) and gave advice that could help not only with athletic performance but also in life.

Every single day, we'd go into the circle before practice, and our coach talks to us and gives us either a quote of the day or something positive to talk about before we head into practice. And, every single time I'm thinking about it, she always like always says, it's not going to just help you right now. But it will help you in your future. (ID# 1088)

Coaches could also be perceived as negative influences who punished players and occasionally created negative feelings towards sport that lasted beyond athletes' retirement.

But my coach didn't always tell me the truth in what he was going to have me do on the team. … it was not what I thought it was going to be. And then after my second year, we had a coaching change. And things got better from there. But I think at that point, my view was kind of already tainted. (ID# 1091)

And we got punished for like a week. And that was the most miserable thing I've ever been through. And I think that probably started the fear of messing up, because I didn't want to be punished physically and get in pain. Stuff like that. We had a lot of [that]. Our coach liked punishing the team; he liked being heavy handed, if you will. And there were a lot of instances where, you know, he would get us up at 2 or 3 AM because someone messed up the night before; we would all go down to the field and run. Or one time he just got really angry, sent us all down in the field and then said just go home. He wanted to just wake us up and get us down there and get us uncomfortable. (ID# 1105)

Athletes interacted with coaches that could help or at times hinder them. Additionally, teammates provided the strength to persevere and somewhere to turn when athletes needed pressure relief. These resources can provide great strength that may be lost as teammates and coaches move away and as athletes retire.

#### Skills and characteristics of student-athletes

Experiences while on a college athletic team led to developing social and leadership skills and personal management abilities. Varsity college athletes were immersed in various settings in which they became skilled at interacting with diverse people, dealing with conflict, and working to improve each other. Athletes recognized their college opportunities would lead to future success.

I was put onto a team with completely different people, everyone from different countries, different backgrounds, different beliefs, different personalities, and there's always conflict. So, we're all here to win. And we're all here for the same purpose and get each other better. (ID# 1016)

Another participant noted skills gained as an experienced leader, after “*receiving the honor of being a captain, the other abilities came later, I'd say being a better public speaker, being better at checking in with people, and listening and guiding, and things like that*” (ID# 1073).

Athletes also talked about how their team experiences facilitated developing discipline including managing time, responsibility, and decision-making:

What's your choice? The personal responsibility of, yeah, the track team will tell you what to eat, when to eat, what do you have to do at this time, but the rest of it, when you go to bed, how you study, the rest of it is all on you. (ID# 1095)

Many talked about their athlete identity and passion for sports and their motivation to set themselves up for success: “*So, invest four years of my life in soccer, because it was my passion but also in something that could give me a future*” (ID# 1008).

#### Changed routine: the COVID-19 pandemic

The changes to routine brought on by the COVID-19 pandemic brought positive and negative influences during athletes’ college careers. The pandemic helped some athletes by giving them an extra year of playing their sport or giving them some time off to recuperate before the next season. Others struggled with their mental health during this time which affected their preparation for continuing competition; access to psychological services was often limited during this time. On a positive note, athletes were often able to be with their teammates and engage in structured training while nonathletes remained more isolated, which athletes surmised would have been even more difficult.

### Theme 2: Transitioning to life after college sport

The highly scheduled lives of college athletes paved the way to a drastic post-retirement loss of structure and support and a changing identity. The transition negatively impacted many, particularly those without a clear post-college sports plan (e.g., graduate school), knowledge of how to manage well their physical health (e.g., exercise, nutrition, prior injury), and strong and supportive social networks.

#### Changing identity and loss of structure

Athletes spoke of the difficult reality of leaving their sport and determining how to cope after collegiate sports and how this impacted their athlete identity.

I understand how this transition works and … I would love to play soccer, but there is a certain period of time that you got to make a decision for your own good, for your own career and for your future. You know? (ID# 1008)

The identity in and comfort with sport was such that some might not take the risk to leave. One athlete mentioned how she watched two friends choose to go back to what they knew instead of going on to a new career.

They keep falling back on being a softball player as who they are. When, you know, one of my friends had a job—and it was a really great job—something that she studied for the whole time she was there. She got her master's in it. But then she went back and now she's coaching, which I think she's a great coach. But I think that's like the main problem that I've seen. You're closing off one door, because you want to fall back on what you know so well. I think that removes some form of personal challenge or growth, because you're just falling back onto something that you did, because it's really comfortable. (ID# 1091)

As their athlete identity was changing, former college athletes also were dealing with major life changes. Having spent much of their school years in sport with a very structured training schedule and structured lifestyles they missed the schedule and routine. After sport they had to learn to adapt to a freedom in which they set their own schedule and made their own lifestyle choices including what to eat, when to exercise, and how to balance sleep and studying or work.

I played five years, I came a semester early too, so like five and a half at [college]. And like every minute's kind of broken down for you exactly what you do, where you need to be when you're working out when you don't want to work out. So then when you have that freedom, I just kind of took a step back and let myself take a break from it. And then the break kind of got a little too long for me. And I was like, okay, I need to have—I like discipline in my life. So, I need a little bit more routine and discipline and finding that. But then also finding it where, like I said, it doesn't need to be workouts that make you want to throw up because you're working out so hard, it can be like a healthy. It was almost like finding a healthy relationship with running again, where it wasn't used as a punishment now anymore, it was used as something that I like to do to feel good. And I like to have a sense of wellness. (ID# 1042)

The transition has been way harder than I anticipated. Just because I'm a very routine guy, disciplined guy. Or when I wake up in the morning, I want to know exactly what I'm doing for the rest of the day. I don't, but right now, I don't have anything going on. I just have school and school's online, and it's very much on my own. (ID# 1134)

I've been less motivated to do things not like on that schedule of like, when you're in sports, they send out a schedule, like you have lift at this time and practice at this time, where now it's up to me to kind of make those schedules. And it's been a lot more difficult to like keep up with because I just am not as motivated to like, go to the gym or like walk… in sports, you had to be there, like, had to do all these things. So, I'm definitely not where I was when I was playing. (ID# 1002)

The lack of structure created a desire for structure in exercise routine.

I miss that, like, team environment, and I miss—with sports, like the constants, the consistent schedule. And so that's one thing I really liked about even just like college athletics is like, alright, you have practice, from this time to this time, every day, you're going to be working out six days a week, like, you don't have a choice where now it just takes a little bit more personal responsibility to get through it. (ID# 1096)

#### Physical health, exercise, and nutrition during the transition

Athletes commonly mentioned feeling physically better after completing collegiate sports including feeling a period of recovery in which they noted less fatigue and aches and pains: “*My body three months later has just recovered a lot more. I've been very keen about getting sleep and actually getting proper recovery* *…* *So, my body definitely feels a lot better now*” (ID# 1143). Athletes noted that they were able to treat these aches and pains with rest as they transitioned after sport.

I'm like in a position where if I, like, wake up in the morning, and like, that hurts really bad or something, I just either like modify my workout, or I don't work out or you know what I mean. And then the same thing, my knee had been bothering me for years, in my career. So, it's been nice to be able to focus on trying to get that better. (ID# 1045)

I was always working towards getting back on the field, but now I'm working towards just for myself, so that's why it's different. But I think those times off from the field with the injury kind of prepped me for now. (ID# 1157)

Some noted how injuries continued to impact their post-retirement daily lives. Without the expertise of an athletic trainer or physical therapist, former athletes were forced to adapt to their injuries without support.

It's [my kneecap has] only dislocated a couple months ago when I was reaching for eggs in the grocery store, like just out of nowhere, just we're done, I guess. Yeah, it's affected daily life where it's like, is it gonna just dislocate now. I don't know, it could. Doesn't matter if you're careful or not. Roll the dice and see. (ID# 1095)

Many noted unhealthy dietary habits and wanting nutritional guidance. Some former college athletes talked about how it took them some time to realize they needed to change their diet and decrease their energy intake in response to lessening exercise.

I'm eating a lot less. Initially, I was eating the same amount. And I just felt sluggish because it was just sitting with me. It wasn't like I wasn't using it and burning it. So, I'm eating a lot less. (ID# 1124)

While a few mentioned their diets were based on good nutrition learned from families, many mentioned how they had not been taught about nutrition sufficiently during college and had to figure it out on their own afterwards.

There was a little bit that people would talk about in college, but that wasn't as large of a focus at my school, and kind of, at least with our program, maybe some other sports were a little different, but it was just kind of a like, you need to eat healthy kind of, but, yeah, I think that really just kind of growing up and how my family was and how we ate, that kind of set the standard. (ID# 1148)

But yeah, it was like, what do I eat and still feel healthy and good because that food was making me feel good. But it's boring. I've been eating the same types of meals for however many years. So that was hard to know how to eat after (ID# 1076)

Some programs had good resources for nutrition and athletes were not prepared to take on their own meals after sports.

Making meals has been hard for me just because while you're in season and while you're working with like, the sports, they give you lunch, they give you dinner, they give you all these things. (ID# 1002)

Athletes also talked about struggling with body image after sport, particularly losing muscle definition and gaining weight.

#### Intersection of mental health and life post-college sport

After sport, athletes frequently noted the psychological stress of the change from athlete with strong team bonds to the individual having to navigate their own path. Participants also talked about changes in friendships, losing the close-knit comradery of the team and having to figure out how to relate to people outside of the sport that had kept them so occupied in college. They talked about how their teammates were people they could reach out to at any time of day or night for the emotional support they needed and some felt these close ties continued. Athletes often had to learn how to navigate friendships outside of the team. “*I have to be more purposeful, about seeing people and doing the things that I want to do*” (ID# 1077).

Some talked about the stress of being a competitive player weighing on them heavily in college and they were glad to be without that pressure.

Now, I'd still consider myself an athlete, but I feel happier, definitely now than I did playing soccer. I would say I'm not trying to rip on the soccer years like they were bad, because they weren't, and there were so many good times. (ID# 1147)

Having a definite plan for life after sport eased the transition for some.

If I didn't know what I was going to do after soccer in college was done, I think it would have been definitely more stressful for me, because if I feel like, in general, if I don't have structure, I'm kind of like, all over the place. I need to set some routine, something like that for me. (ID# 1147)

### Theme 3: Empowering former athletes to live more healthfully

Athletes voiced many comments about how they could have been empowered to live more healthfully in the transition away from college athletics, although many comments were expressed in terms of needs or challenges during the transition period (see Theme 2 above).

#### Resources to facilitate transition

Some expressed how helpful it would be to have formal assistance with the transition, such as information packets, a transition team, regular seminars, or classes. “*Maybe somebody could facilitate some sort of study hour and then that's dedicated for the athletes that are going to be graduating to just start thinking about what it is you want your next phase to look like*” (ID# 1091). Athletes recommended topics such as athlete identity, nutrition changes after sport, strength and conditioning based on post-sport goals, dealing with long-term injuries, and finances. The suggested frequency and delivery methods varied.

My dream goal would be when you are done with college athletics, at the minimum … you're given a packet of supplementary information. Instead of just, you're done, see you later. And that would be nutrition tips and lifting tips … having some type of team to help facilitate that transition. (ID# 1076)

Post-retirement athletes wished for continued access to support services including athletic trainers, dieticians, physical therapists, and physicians.

As soon as you're done, you're done like, you know what I mean? Like you lose access to a lot of things… but I think letting you hang on to [specific resources] for like, a couple months maybe could be beneficial in that transition. (ID# 1045)

My girlfriends who have transitioned out… gained weight after they were done and they want to lose weight and it's not healthy, don't have a good relationship with exercise. [They] over exercise or, you know, don't eat enough and just starve themselves. And so, knowing the resources in your community to help with things like that and also being aware of those things. (ID# 1076)

I actually tweaked my back a little bit, I think last week or two weeks ago. So again, it's just kind of trying to find ways to adjust. I mean, I don't have access to an athletic trainer anymore, which kind of sucks, but I still have all those rehab exercises. (ID# 1148)

While feeling physically better as they recovered from competitive sport, athletes desired more structure and advice for managing prior injuries and setting a plan for balance eating and physical activity: “*It’d be awesome if we had this team of nutritionists and personal trainers who could help those football players transition in those next six months before they graduate into just a healthy lifestyle*” (ID# 1076). Athletes desired specific plans for how to fit exercise into life.

Just figuring out what work life looks like, what different ways I can still stay active and keep playing volleyball … figuring out where I can keep volleyball in my life, where I can keep kind of still like lifting, trying to figure out some of that, trying to finally work in cardio a little bit in my life. Because that was never a focus. I was always playing volleyball enough in college that I kind of just counted that and throughout the workout. So, you know, I'm just figuring out what new life looks like, and it's an adjustment. (ID# 1148)

Participants also expressed the need to talk to someone about life after competitive sports, particularly the impact on their mental health.

Maybe letting yourself talk to people about it [the transition] could help and even just knowing they felt the same way would be comfortable, comforting. And just like asking them those questions of like, oh, what do you do for your workouts now? Or like, how has your diet changed and stuff like that, I think could be beneficial in the initial moment of like, what do I center my life around now when the thing you centered your life around is gone (ID# 1045)

You're not ready for it [the transition], dude, you're just not because it's different not being able to keep that same … regimen that you've been doing for your whole life…You just kind of get used to it. So I'd say talk to somebody during those first four months afterwards because you're not going to be ready for that transition into a nonathlete. (ID# 1095)

Most thought the person to talk to should be someone who had experienced the transition, perhaps a coach, or a former athlete, or in some cases a counselor or sport psychologist, particularly one who had been an athlete. Like retaining physical health resources during the transition period, some participants noted that continued access to their sports psychologist would have been helpful.

I think a sports psychologist would have made a difference. Have they had experience in sport? Because it would have been really tough to trust him and to relate to him, or her to just to relate to them had they not also experienced it. Because if they were just a sports psychologist, but only coming from a psychology background, then I would always there would always be that that twinge in the back of your mind. That's like, you don't actually know what it's like to be me do you. Which is why like, if me now could have talked to former me, it would have been really helpful. (ID# 1035)

However, participants noted that some athletes may not be mentally ready to talk about what was coming after competitive sport during their sports careers, even in their final season. While admitting, “*At the same time, like when you're in your last season, you don't really want to talk about what it's gonna look like afterwards, you just know that it's like going to be hard*” (ID# 1045). Another admitted they spent so much time in sport they felt unskilled in cooking healthy easy meals and despite needing some *“dialogue about that. I don't know if I would have done this personally, if it was offered to me. I don't think I would have, but I think it could help some people like transitioning mentally” (ID# 1015).*

#### Career planning

Athletes also felt the need to be empowered through career planning assistance. One commented, “*I think I needed to establish relationships with faculty earlier. Other than that, I'm a pretty independent person in general. So, kind of finding those resources myself has kind of been second nature to me since the beginning*”. (ID# 1002).

Having spent years in successful and high achieving athletic careers, they were apprehensive about being career novices.

I knew that I could be a dominant player in, you know, in college, while once I finished school, I always be a normal, decent student. So, I just want to challenge my knowledge with, you know, people that have a lot of experience. I'm just concerned about their judgment, because I think I tried to get inspiration with people that are better than me. So, I will be disappointed if this will go well, you know, if I will have a better relationship with somebody that has more, you know, experience knowledge than me in my field. (ID# 1008)

#### Advice

Former athletes advised athletes on various topics. They wanted athletes to love their sport and to recognize that it is not all about the goals, but rather how important it is to enjoy the journey.

Don't fall out of love with your sport, have your goals but also have fun. You need to trust the process. Enjoy the journey and stop and smell the roses. That's one thing athletes suck at doing is giving yourself a pat on the back. Right? You achieve a goal. Okay, what's next? (ID# 1043)

Enjoy every minute of it. I mean, you never know when it's gonna end. You don't know if you I mean, someone could get hurt tomorrow … so I mean treasure the friendships you've made. Love the people that you're around every day and keep up with it. You know, if you surely value, the friendship you'll keep talking to them more than just a couple weeks after parting ways. (ID# 1058)

Former college athletes also wanted athletes to recognize there was more than sports in life. As sports ended, one athlete stated “*I don’t remember any of the records* *…* *it's all the other stuff. It's all the time in between, time off tasks. That's going to be the stuff that you remember*” (ID# 1153).

Another athlete advised to not let “*your sport take over your life. Which is, like I said, it's like so difficult when that like it honestly is your life. And I don't know how you don't make it your life*” (ID# 1013).

## Discussion

This study probed how the unique experiences of college varsity athletes impacted the transition away from sport and identified facilitators, barriers, and needs of college varsity athletes to engage in a physically active and healthy lifestyle after competitive sport retirement. College athletes have unique experiences that both equip and challenge them as they transition away from structured sport environments and the associated support systems. Strong support systems are often lost at the time of separation from sport. Young adult former college athletes identified several key factors that may facilitate a healthier transition, including guidance on exercising for general health, managing pain and prior injuries, nutrition, and social support (Table 3). Understanding the needs of athletes transitioning out of competitive sport will better equip healthcare providers to counsel, educate, and treat athletes for optimal long-term health.

**Table 3:** Key opportunities to help facilitate athlete health and wellness during and after the transition away from competitive sports.

Category	Goal(s)	MVPs (key people)	Tips/notes for athletes
Injury & Recovery Zone	Manage prior injuries and pain	Primary Care Provider Sports Medicine Physician Physical Therapist Athletic Trainer	– Create a recovery plan – Continue to receive needed rehabilitative services
Redefining Fitness Goals (Post-Sports)	Set new fitness goals	Personal Trainer Health Coach Recently Graduated Former Athletes Coaches	– Build a fitness plan – Find workout partners – Share community activity resources with other athletes – Meet up at parks
Physical Activity and Exercise	Exercise for general health and establish fun activities	Athletic Trainer Physical Therapist Personal Trainer	– Join a recreational sports league – Set daily step-count goals – Discover a new sport – Explore nature
Nutrition Plan	Learn to create a healthy meal plan for new activity level	Dietician	– Attend community cooking classes – Start a recipe swap with other former athletes
Social Support	Foster “team vibes” through relationships	Former Teammates Alumni Networks New Organizations	– Foster new relationships – Get involved with groups – Develop a new ‘team’
Mind Matters (Transition Timeout)	Support mental health during transition	Psychologist Former Athletes Sport Alumni Network Lifestyle Coach	– Engage in alumni events – Join a community sports team – Discover a new passion (e.g., music, art)
Career Kickoff	Prepare for career success	Mentors Career Counselor Financial Advisor	– Establish regular meetings with a mentor or advisor – Create career advancement goals – Recognize athletic success in meeting goals as you plan career success
3–6 Month Support Plan: – Continued access to facilities and sports medicine resources – Guidance on exercising for general health (rather than sport performance) – Nutritional guidance and meal planning and prepping resources – Mental health check-ins and peer support groups – Career planning and mentoring			
**A Balanced, Healthy, Purposeful Life After Sports**			

The first theme, college athlete uniqueness, emphasized the distinctive benefits and challenges faced by collegiate athletes. College athletes live scheduled and structured lives that can be demanding and rewarding, humbling and glorifying, socially rich and supportive yet uniquely challenging, and filled with perks and pressures few others face. These unique experiences shape a framework that has long-lasting implications that can be both positive and negative. Team experiences lead to strong social and leadership skills. Athletes connect closely with peers and coaches, which may lead to lifelong friendships. Athletes can experience physical and/or mental health challenges. The demanding schedule and stress of competitions may lead to burnout. Yet, the same stressors of sport can also be a place to relieve worries such as those caused by academic demands. Athlete identity encompasses lives and is often challenged by changes due to injury or the transition into retirement, consistent with prior literature (4, 11, 26–28).

The second theme, transitioning to life after college sport, highlighted the difficulties of losing the strong structure and support following retirement from college athletics. While many assume that (former) athletes typically remain active, this is not always the case, and former athletes may face barriers that are similar to and different than nonathletes. Ferrara et al. interviewed 17 former college athletes (age range: 22–33 years old) who currently did not meet physical activity guidelines and described barriers to engaging in exercise after retiring from college sports including lifestyle changes (e.g., new life responsibilities), loss of motivation, cost, lack of resources, lack of time, and physical limitations holding them from maintaining a physically active lifestyle (29). Our current study identified some challenges and opportunities to overcome barriers to engaging in exercise and physical activity as discussed in theme 3. Similar to findings from our prior study in midlife (age 51 ± 7 years) former collegiate athletes (4), the present study indicates that concerns regarding managing pain and prior injury exist in young athletes too. There may be an opportunity to intervene prior to or around the transition period of sport retirement to address these difficulties earlier.

Another related barrier to physical activity and exercise that merits further consideration in future research is the relationship with exercise that athletes learn through sport. Notably, athletes mentioned needing to develop “a healthy relationship with running again” since running is a common form of punishment in sports. It is possible that some athletes develop a negative relationship with running and other aerobic exercise that is important for long-term physical activity and health due to its association with punishment. Future research should explore avenues that help former competitive athletes overcome this and other barriers associated with long-term engagement in and enjoyment of running and other forms of aerobic exercise.

The intersection of physical and mental health challenges during and following sports were also prominently featured in our findings which corroborates prior research (10, 26, 30, 31). While the present qualitative study and ongoing parent clinical study focus on physical health and function, we would be remiss in not acknowledging the complex and intertwined physical health (including injury/pain status, and current fitness/exercise levels) and mental health (including interpersonal relationships) of retired college athletes (4, 32). A systematic review and meta-analysis by Mannes et al. found similar rates of psychological distress (depression, anxiety, substance misuse, etc.) in retired elite athletes compared with the general population, although there was considerable variation among subgroups: prevalence of depression ranged from 4.7% to 29% and anxiety ranged from 8.4–16.2%, which was highest among former NCAA Division I collegiate athletes (30). A more recent scoping review of existing reviews and programs for adaptation to life after sport for retired athletes by Voorheis and colleagues concluded that most reviews found high prevalence of mental health issues, including anxiety, depression, and alcohol misuse, among retired athletes (28). Similar to findings from a qualitative study in 21 NCAA Division II athletes who discontinued their sports, some athletes—particularly those who did not complete their full eligibility—in our study experienced similar themes of stress, burnout, and pressure (12). Yet unlike that study's sample (12), most participants in our study completed their eligibility and expressed many positive experiences from their college sports participation as well. Ludqvist et al.'s small qualitative study in Swedish elite athletes highlighted the need for sports organizations to ensure that psychotherapy resources are accessible (33). Our study also supports the notion that psychotherapy resources are often insufficient in the collegiate athlete setting (dependent on division and sport) and are typically absent once an athlete retires from college sport—echoing a need described by Reifsteck and colleagues (19). The interrelated physical and psychosocial challenges that retiring athletes face in their transition to life after competitive sport point to a need for an integrated and comprehensive transition plan.

The third theme, empowering former athletes to live more healthfully, captured athletes' comments on what could have made transition easier. Young adult former college athletes identified several key factors that may facilitate a healthier transition, including guidance on exercising for general health, managing pain and prior injuries, nutrition, and social support (Table 3). Facilitators to engage in a more healthy and active lifestyle post-college sports include receiving education on exercising for general health instead of athletic performance; having additional resources such as access to a personal trainer to facilitate exercise planning; overcoming pain and prior injury which could be facilitated by ongoing support from health professionals such as an athletic trainer or physical therapist; determining physical activity opportunities and goals post college sports which mentoring relationships with older former college athletes or coaches could help; and peer support groups to continue to benefit from the social aspect of exercise, sports, and teams. While athletes generally agree that additional support is needed, various opinions exist about the optimal timing, methods, content, stakeholders involved, and prioritization of resources and support. Understanding the needs of athletes transitioning out of competitive sport will better equip healthcare providers to counsel, educate, and treat athletes for optimal short- and long-term health.

### Limitations

There are both strengths and limitations to consider when interpreting the findings. The sample size (*n* = 30) is larger than most qualitative research studies and included equal representation of male and female athletes, although comparisons between males and females were beyond the scope of this study and should be considered in future works. Although most athletes played NCAA Division I sports (87%), some representation from NCAA Division III and NAIA athletics was present. Participants represented a wide distribution of sports although not every collegiate sport was represented. An inherent strength of open-ended qualitative methodology is gathering large amounts of information and breadths of experiences, though considerable variability of viewpoints was noted as discussed above. Furthermore, the findings of this study should be interpreted within the context of when the participants were in college—amidst an unprecedented pandemic due to COVID-19 and a rapidly changing landscape of professionalism within collegiate sports due to the name, image, and likeness (NIL) adoption in July 2021.

### Future directions

While this qualitative study helps to address some of the research needs highlighted by Reifsteck et al.'s (19) call to promote student-athlete well-being during the transition from collegiate sport, our findings also underscore the unmet needs of collegiate athletes transitioning to their next phase of life. Athletes experience an abrupt shift from structured schedules and substantial support to a lack of both. There is a great need for more mixed-methods research, such as the protocol described by Ronkainen and colleagues (34) and our ongoing work. While former collegiate athlete participants in the present study agreed that additional resources and support would be beneficial, there were differing opinions on the optimal timing (e.g., during and/or after their college athletic careers), methods, content, stakeholders involved (e.g., former athletes, coaches, athletic trainers, physical therapists, personal trainers, psychologists, dieticians, etc.), and prioritization of resources and support. Future research should develop and test interventions to address the ongoing needs of collegiate athletes as they transition to life after competitive sports to position them best for long-term health and wellness.

## Conclusion

Competitive athletes have unique experiences that both equip and challenge them as they transition away from structured sport environments and the associated support systems and into life after sport and after college. Former athletes identified several key factors that may facilitate a healthier post-collegiate sport transition, including guidance on exercising for general health, managing pain and prior injuries, nutrition, and mental health support. While athletes generally agreed that additional support is needed, they discussed various opinions about the optimal timing, methods, content, stakeholders involved, and prioritization of resources and support. Understanding the needs of athletes transitioning out of competitive sport will better equip healthcare providers to counsel, educate, and treat athletes for optimal long-term health and ultimately help empower (former) athletes to live more healthfully.

## Data Availability

The data for the relevant findings are included within the manuscript. The interview question guide was previously published (4) and is available upon request. Due to the nature of qualitative research and concerns over privacy and participant anonymity, complete transcripts of data are not available.
